# Severe Dengue Is Associated with Consumption of von Willebrand Factor and Its Cleaving Enzyme ADAMTS-13

**DOI:** 10.1371/journal.pntd.0001628

**Published:** 2012-05-01

**Authors:** Kis Djamiatun, Andre J. A. M. van der Ven, Philip G. de Groot, Sultana M. H. Faradz, D. Hapsari, Wil M. V. Dolmans, Silvie Sebastian, Rob Fijnheer, Quirijn de Mast

**Affiliations:** 1 Department of Parasitology, Faculty of Medicine, Diponegoro University, Semarang, Indonesia; 2 Department of General Internal Medicine, Radboud University Nijmegen Medical Centre, Nijmegen, The Netherlands; 3 Laboratory for Thrombosis and Haemostasis, Department of Clinical Chemistry and Haematology, University Medical Centre, Utrecht, The Netherlands; 4 Division of Human Genetics, Faculty of Medicine, Diponegoro University, Semarang, Indonesia; 5 Department of Pediatrics, Faculty of Medicine, Diponegoro University, Semarang, Indonesia; Tropical Medicine Institute Pedro Kourí (IPK), Cuba

## Abstract

**Background:**

Thrombocytopenia, bleeding and plasma leakage are cardinal features of severe dengue. Endothelial cell activation with exocytosis of Weibel-Palade bodies (WPBs) may play an etiological role in this condition.

**Methods and Principal Findings:**

In a cohort of 73 Indonesian children with dengue hemorrhagic fever (DHF), of which 30 with dengue shock syndrome (DSS), we measured plasma levels of the WPB constituents von Willebrand factor antigen (VWF:Ag), VWF propeptide and osteoprotegerin (OPG), together with activity levels of the VWF-cleaving enzyme ADAMTS-13 and the amount of VWF in a platelet binding conformation (VWF activation factor). Compared with healthy controls (n = 17), children with DHF/DSS had significantly higher levels of VWF:Ag, VWF propeptide and OPG and decreased ADAMTS-13 activity. The VWF activation factor was also significantly higher in DHF/DSS and highest in children who died. There were significant differences in the kinetics of the various WPB constituents: VWF propeptide and OPG levels decreased toward discharge, while VWF:Ag levels were lower than expected at enrollment with plasma levels increasing toward discharge. Moreover, VWF propeptide levels correlated better with markers of disease severity (platelet count, liver enzymes, serum albumin and pleural effusion index) than corresponding VWF levels. Together, these findings suggest that there is consumption of VWF in DHF/DSS. In 4 out of 15 selected children with low ADAMTS-13 levels on admission, we found a remarkable reduction in the large and intermediate VWF multimers in the discharge blood samples, consistent with an acquired von Willebrand disease.

**Conclusion:**

These findings suggest that severe dengue is associated with exocytosis of WPBs with increased circulating levels of VWF:Ag, VWF propeptide and OPG. High circulating levels of VWF in its active conformation, together with low ADAMTS-13 activity levels, are likely to contribute to the thrombocytopenia and complications of dengue. During the convalescence phase, qualitative defects in VWF with loss of larger VWF multimers may develop.

## Author Summary

Severe dengue infections are characterized by thrombocytopenia, clinical bleeding and plasma leakage. Activation of the endothelium, the inner lining of blood vessels, leads to the secretion of storage granules called Weibel Palade bodies (WPBs). We demonstrated that severe dengue in Indonesian children is associated with a strong increase in plasma levels of the WPB constituents von Willebrand factor (VWF), VWF propeptide and osteoprotegerin (OPG). An increased amount of the hemostatic protein VWF was in a hyperreactive, platelet binding conformation, and this was most pronounced in the children who died. VWF levels at enrollment were lower than expected from concurrent VWF propeptide and OPG levels and VWF levels did not correlate well with markers of disease severity. Together, this suggests that VWF is being consumed during severe dengue. Circulating levels of the VWF-cleaving enzyme ADAMTS-13 were reduced. VWF is a multimeric protein and a subset of children had a decrease in large and intermediate VWF multimers at discharge. In conclusion, severe dengue is associated with exocytosis of WPBs with consumption of VWF and low ADAMTS-13 activity levels. This may contribute to the thrombocytopenia and complications of dengue.

## Introduction

Dengue has become a major international public health concern with up to 100 million annual cases worldwide. It usually manifests as a non-specific febrile illness, but its course may become complicated by bleeding and a transient plasma leakage that may ultimately lead to shock and death [1]. Severe dengue with thrombocytopenia, bleeding and plasma leakage is referred to as dengue hemorrhagic fever (DHF). The most severe form of DHF, which is accompanied by hemodynamic instability and shock is referred to as dengue shock syndrome (DSS). DHF/DSS is most frequently seen in children and tends to manifest at the time the fever subsides. The pathogenic mechanisms responsible for the development of DHF/DSS are still poorly understood. A central feature of DHF/DSS is the development of a pronounced thrombocytopenia.

The large glycoprotein von Willebrand factor (VWF) plays a central role in platelet-vessel wall interaction as it is responsible for mediation of platelet adhesion at sites of endothelial injury. VWF is predominantly synthesized in endothelial cells and, after cleavage of a VWF propeptide, it is either released constitutively or stored in specialized secretory granules, known as Weibel-Palade bodies (WPBs). Injury or activation of the endothelium leads to a rapid secretion of equimolar amounts of stored VWF and VWF propeptide, and both proteins are regarded as markers of endothelial cell activation [2]. Freshly released VWF consists of ultra-large prothrombogenic multimers (UL-VWF). The metalloprotease ADAMTS-13 (a disintegrin and metalloproteinase with thrombospondin-1-like domains) functions as a natural regulator that de-activates the prothrombogenic UL-VWF by proteolysis [3]. The importance of ADAMTS-13 is illustrated by the notion that absence of ADAMTS-13 is associated with platelet-rich microthrombi in the microvasculature, a disease known as thrombotic thrombocytopenic purpura (TTP) [4]. Under normal circumstances, VWF circulates in the plasma in a knot-like conformation in which the binding sites for platelet glycoprotein (GP) receptor Ib is not exposed. A change in conformation into a more elongated ‘activated’ form can be observed under different conditions in which increased platelet-VWF interaction is thought to play a role [5]. Determination of the amount of this ‘active’ VWF in plasma is possible using a llama-derived nanobody (designated AU/VWFa-11) that displays specific binding only to the GPIba-binding conformation of the VWF.

Osteoprotegerin (OPG) is a member of the tumor necrosis factor receptor superfamily, which is stored in WPBs and in platelets [6], [7], similar to VWF. OPG was traditionally known for its role in bone remodeling, but a growing body of literature suggests that OPG also has specific effects on the endothelium, including stimulation of vasculogenesis [8], expression of adhesion molecules [9] and adhesion of leukocytes [10]. Moreover, OPG is physically associated with the A1 domain of VWF, both in stored and in circulating VWF, suggesting that OPG could interfere with platelet-VWF binding [11].

Children with an acute dengue infection have elevated VWF levels [12], [13] and one study reported a moderate decrease in ADAMTS13 activity using an indirect assay for ADAMTS13 activity [14]. The aim of this study was to characterize WPB exocytosis and changes in factors involved in VWF-platelet interaction in patients with severe dengue. We determined concentrations of VWF antigen (VWF:Ag), VWF propeptide, OPG and ADAMTS-13 activity in serial obtained plasma samples from a cohort of Indonesian children with DHF/DSS, together with the multimeric pattern of VWF and the amount of VWF in a platelet binding conformation (VWF activation factor).

## Methods

### Ethics statement

The Research Ethics Committee of the Faculty of Medicine Diponegoro University, Semarang, Indonesia, approved all legal, ethical and laboratory aspects of the study. Written informed consent was obtained from parents or legal guardians of the patients.

### Patients and study procedures

This observational study enrolled consecutive children aged 3–14 years who were admitted to the pediatric ward or intensive care unit of the Dr. Kariadi University Hospital in Semarang, Indonesia between July 2005 and July 2006 with a clinical diagnosis of suspected DHF/DSS according to the 1997 WHO criteria [15]. A control group of 17 healthy Indonesian children, aged 6 to 14 years, was also enrolled. Inclusion criteria and clinical characteristics for this group have recently been described [16]. All patients tested positive for a dengue specific IgM in the discharge blood sample. DSS was defined as DHF with evidence of circulatory failure.

Blood samples were collected in citrate anti-coagulated blood tubes (Beckton-Dickinson) at hospital admission (enrollment; day 0), on day 1 after admission and on day of discharge. A full blood count was performed daily in all patients as part of routine clinical care until platelet counts had shown a substantial increase. Therefore, no platelet counts or other hematological values were usually available on the day of discharge. A chest X-ray was performed with the patient lying in right lateral decubitus position to detect pleural effusion at enrollment and on day 2. The pleural effusion index (PEI) was calculated by dividing 100 times the maximum width of the pleural effusion by the maximum width of the hemi-thorax.

### Laboratory procedures

Citrate blood was centrifuged for 20 minutes at 1600 g and plasma was stored at −80°C until further analysis. Plasma levels of VWF:Ag and VWF propeptide were determined by enzyme-linked immunosorbent assay (ELISA) as described previously [17]. Active VWF was determined by ELISA using a nanobody (AU/VWFa-11) that specifically recognizes the GP-1bα binding configuration of VWF, as described previously [18]. The term VWF activation factor was used to express the relative amount of VWF that circulates in its active, platelet binding conformation. VWF activation factor of normal pooled plasma was referred to as 1. The multimeric pattern of VWF was analyzed in a selection of 15 patients, among whom the patients with the lowest ADAMTS-13 activity levels at enrollment or on day 1 using 2% agarose gel electrophoresis, followed by in-gel immunostaining and infrared imaging [19]. ADAMTS-13 activity was determined using the fluorescence resonance energy transfer (FRETS) assay for ADAMTS-13 activity (Peptides International, Inc., USA) whereby the ADAMTS-13 activity of normal pooled plasma (NPP) of healthy Dutch donors was set at 100% [20]. Values obtained in the study participant samples were expressed as percentage of NPP. Normal values in healthy volunteers in our laboratory were in the range of 60–140%. OPG was measured by an in-house ELISA using microtiter plates coated overnight at 4°C with a mouse anti-human OPG antibody (MAB8051, R&D Systems, Minneapolis, MN). Diluted plasma samples were incubated for 2 h at room temperature. Detection was done by incubation with a secondary goat polyclonal antibody (BAF805, R&D Systems, Minneapolis, MN) and streptavidin-conjugated HRP (Sanquin, Amsterdam, the Netherlands) and SuperSignal West Pico Chemiluminescent Substrate (Thermo Scientific). For statistical analyses, the upper limit of detection for OPG (1200 mg/L) was used in samples with values above this detection limit. Serum SGOT concentrations (normal value for 7 year old children ≤35 Unit/L) were measured using a colorimetric method (Hitachi 7050; Boehringer Ingelheim, Germany). Serum albumin concentrations (normal value ≥3.26 g/dl) were measured using Bromocresol–Green method. Presence of dengue specific IgM and IgG antibodies was determined by capture and indirect ELISA (Focus Technologies, Cypress, Calif., USA), according to the manufacturer's instructions [21]. A full blood count was performed daily by a standard hematology analyzer.

### Statistical analyses

Data were expressed as medians with interquartile ranges (IQR). Differences between groups were assessed by Mann-Whitney tests; changes in laboratory parameters over time within groups were evaluated by Wilcoxon matched-pairs signed rank test. Relationships between continuous variables were examined by Spearman's rank correlation analysis. A p-value of <0.05 indicated a significant difference. Statistical analyses were performed with SPSS version 16.0.

## Results

### Clinical characteristics and treatment

A total number of 73 children with severe dengue were enrolled, of whom 43 were classified as having DHF grade I or II and 30 as DSS (DHF grade III or IV). In addition, 17 healthy controls were enrolled. Clinical characteristics of the patients are summarized in table 1 (adapted from [16]). The median duration of fever at enrollment was 4.0 days in both groups. All patients showed plasma leakage on day two after enrollment as evidenced by pleural effusion on a lateral chest X-ray. The nadir in platelet counts was observed on day 1 with median (IQR) values of 45×10^9^/L (27–75×10^9^/L) and 50×10^9^/L (18–74×10^9^/L) for the DHF and DSS group; by day 3, median platelet counts had raised to 89×10^9^/L (43–111×10^9^/L) and 80×10^9^/L (40–153×10^9^/L), respectively. Clinical bleeding occurred in 7 (16.3%) patients with DHF (epistaxis, n = 4; gum bleeding, n = 1; hematemesis, n = 2) and in 2 (7.1%) patients with DSS (hematemesis, n = 1; melena, n = 1). Intravenous fluids were administered to all but three children. Free frozen plasma (FFP) was administered to 3 (7.0%) children with DHF and to 8 (28.6%) children with DSS. Two (4.7%) children from the DHF group and 4 (14.3%) from the DSS group received a platelet transfusion. Six children, all from the DSS group, died during the admission. No plasma sample was available for analysis in two patients from the DHF group and one patient from the DSS group at day 1 and in fifteen and six patients of these respective groups at discharge.

**Table 1:** Patient characteristics and baseline data for children with dengue hemorrhagic fever/dengue shock syndrome.

Characteristic	DHF (DHF I and II)	DSS (DHF III and IV)
	n = 43	n = 30
Male sex, n (%)	15 (35)	10 (33)
Age, years	8.0 (6.0–9.0)	7.0 (6.0–9.3)
Mortality, n (%)	0 (0%)	6 (20%)
Body weight, kg	20 (18–28)	22 (17–30)
Duration fever until admission, days	4.0 (3.0–5.0)	4.0 (4.0–5.0)
Tourniquet test positive, n (%)	26/36 (72)	11/17 (65)
Petechiae per 2.5 cm^2^, n (%)	26 (8)	24 (5)
Hemoglobin, g/dL		
Enrollment	13.1 (12.3–14.0)	13.6 (12.2–14.4)
Day 1	13.0 (11.7–13.6)	12.7 (10.9–13.4)
Platelet count, ×10^9^/L		
Enrollment	64 (41–85)	38 (25–71)
Day 1	45 (27–75)	50 (18–74)
Albumin serum, g/dL		
Enrollment	3.5 (2.9–3.8)	2.8 (2.4–3.3)*
Day 2	3.5 (3.1–3.7)	3.3 (3.0–3.7)
Total protein serum, g/dL		
Enrollment	5.8 (4.7–6.5)	4.8 (3.8–5.7)*
Day 2	6.0 (5.4–6.6)	5.9 (5.0–6.3)
SGOT, U/l		
Enrollment	103 (87–184)	118 (77–199)
Day 2	93 (64–143)	126 (91–224)*
Pleural effusion, n (%)		
Enrollment	25/33 (76)	23/26 (88)
Day 2	39/39 (100)	24/24 (100)
Pleural effusion index		
Enrollment	10.0 (2.0–20.2)	19.4 (12.7–29.4)*
Day 2	24.5 (15.4–32.7)	30.9 (23.1–42.8)*

Data represent median with interquartile ranges or numbers with percentages or number with percentage. DHF, dengue hemorrhagic fever; DSS, dengue shock syndrome; SGOT, serum glutamic oxaloacetic transaminase.

*: p<0.05 by Mann-Whitney U-test.

### VWF:Ag, VWF propeptide and OPG

**Figure 1:**
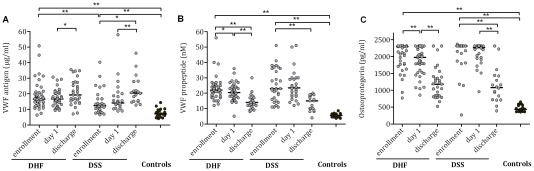
Plasma levels of Weibel-Palade body constituents in Indonesian children with dengue. (A) VWF antigen, (B) VWF propeptide and (C) osteoprotegerin levels (all determined by ELISA) in Indonesian children with dengue hemorrhagic fever (DHF) and dengue shock syndrome (DSS) and in healthy controls. Horizontal lines represent median values. The upper limit of detection of the OPG assay was 1200 pg/ml; 49% and 70% of children in the DHF and DSS group, respectively, had an OPG plasma level above this cut-off value at enrollment. No plasma available for analysis in two patients from the DHF group and one from the DSS group at day 1 and in fifteen and six patients from the DHF and DSS group at discharge. *P* values were determined by Wilcoxon matched-pairs signed rank test for data in time and Mann Whitney U- test for comparison with the control group. *p value<0.05, **p value<0.01.

Children from the DHF group had the highest VWF:Ag levels at enrollment, followed by children with DSS and healthy controls with median (IQR) values of 16.6 µg/mL (12.9–20.6 µg/mL), 12.5 (9.9–16.8 µg/mL) and 7.2 µg/mL (5.8–9.5 µg/mL), respectively (Figure 1A). In contrast, the highest VWF propeptide levels were found in the DSS group with a median level of 22.7 nM (15.7–35.8 nM) compared to 21.9 nM (17.7–24.9 nM) in the DHF group and 5.7 nM (4.7–6.4 nM) in healthy controls (Figure 1B). There was also a clear difference in the kinetics of VWF:Ag and VWF propeptide levels, despite the fact that both proteins are released into the plasma in equimolar amounts. While VWF propeptide levels decreased towards discharge, VWF:Ag levels increased significantly. OPG levels followed the same pattern as VWF propeptide: baseline levels were very high with 49% and 70% of the children in the DHF and DSS group, respectively, having a value above the upper detection limit of the assay (1200 mg/L), followed by a decrease towards discharge (Figure 1C).

### VWF activation factor and ADAMTS-13

**Figure 2:**
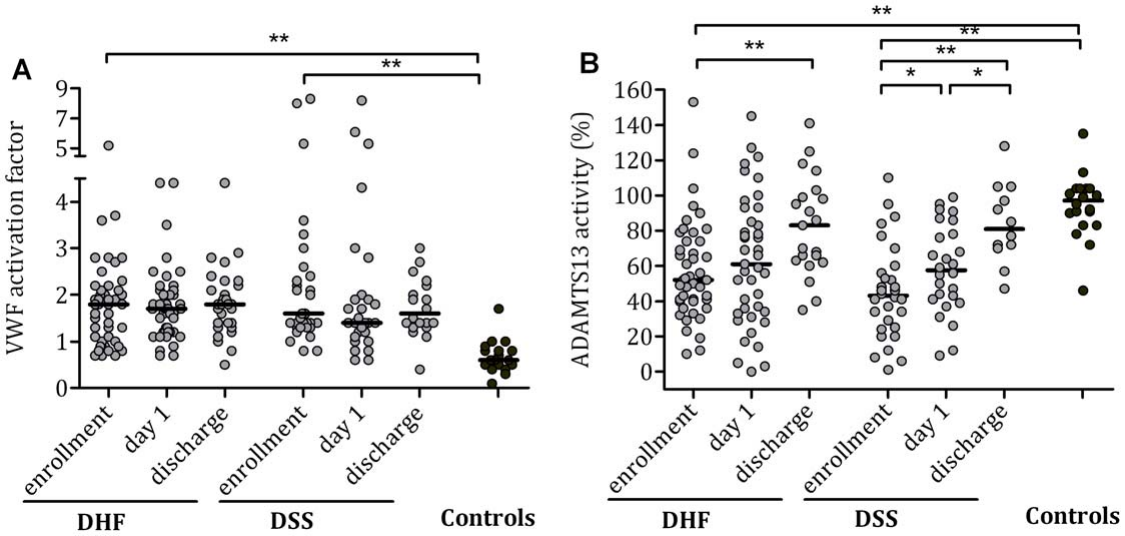
Plasma ADAMTS-13 activity level and VWF activation factor in Indonesian children with dengue. (A) ADAMTS-13 activity level in children with dengue hemorrhagic fever (DHF) and dengue shock syndrome (DSS) and in healthy controls. The ADAMTS-13 activity levels were determined by FRETS-VWF73 assay and are depicted in % of normal pool plasma. (B) VWF activation factors were determined by ELISA using the llama-derived nanobody AU/VWFa-11. The VWF activation factor represents the relative amount of VWF that circulates in an active platelet-binding conformation, whereby the VWF activation factor of normal pooled plasma was referred to as 1. Horizontal lines represent median values. No plasma available for analysis in two patients from the DHF group and one from the DSS group at day 1 and in fifteen and six patients from the DHF and DSS group at discharge. *P* values were determined by Wilcoxon matched-pairs signed rank test for data in time and Mann Whitney U-test for comparison with the control group. *p value<0.05, **p value<0.01.

The median VWF activation factor was about twofold higher in the DHF/DSS patients than in the healthy controls, indicating that a higher amount of the circulating VWF was in a platelet-binding conformation (Figure 2A). This increased activation status of VWF persisted until discharge. A marked reduction in ADAMTS-13 activity levels was also a common finding at the time of enrollment (Figure 2B). At enrollment, 20/43 (46%) of children with DHF and 20/30 (67%) of children with DSS had an ADAMTS-13 activity level of ≤50%; a severe ADAMTS-13 deficiency (≤10%) was found in 1/43 (2%) and in 3/30 (10%), respectively. ADAMTS-13 activity levels recovered to near normal values at the time of discharge.

### VWF multimeric pattern

**Figure 3:**
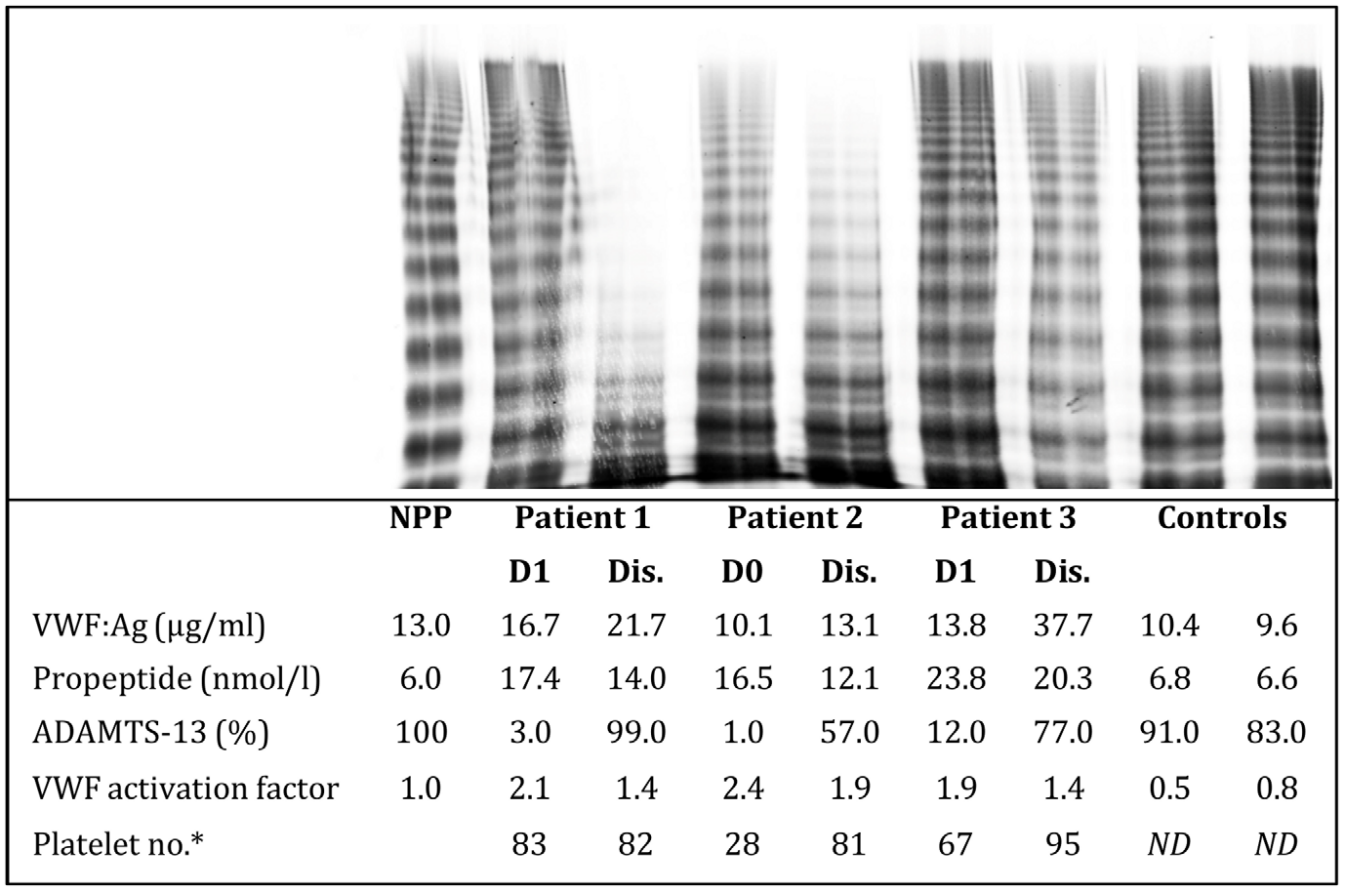
VWF multimer pattern in Indonesian children with dengue. Plasma VWF multimer distribution was analysed by agarose gel electrophoresis, followed by in-gel immunostaining. Electrophoresis was performed from the top to the bottom. Plasma at discharge (Dis) from 3 children with severe dengue (patient 1–3) demonstrated a reduction in large and intermediate VWF multimers compared to plasma at enrollment (D0) or day 1 (D1), consistent with an acquired von Willebrand disease. No ultralarge VWF multimers were seen. NPP depicts normal pool plasma. VWF:Ag, VWF antigen. * The last available platelet count before discharge is given.

The multimeric pattern of VWF was determined in 15 patients. These 15 patients included the 7 patients of the cohort with the lowest ADAMTS-13 activity levels at enrollment in whom a blood sample at discharge was available; the remaining 8 patients were randomly selected. Despite the low ADAMTS13 levels, UL-VWF was not observed in any of the blood samples. However, the discharge blood sample of 4 patients with low ADAMTS-13 levels at enrollment showed a reduction in large and intermediate VWF multimers (Figure 3; patient 1 to 3, patient 4 not shown). None of these 4 patients suffered from clinical bleeding.

### Relation to severity of illness and correlation between parameters

In the group of children with DSS, there was no significant difference between the 6 children who died compared with those who survived in median values at enrollment for VWF:Ag levels (14.9 µg/mL vs. 12.3 µg/mL; p = 0.53), VWF propeptide levels (33.3 nM vs. 21.7 nM; p = 0.10), ADAMTS-13 activity (30% vs. 49%; p = 0.19) and platelet count (37×10^9^/L vs. 58×10^9^/L; p = 0.19). The most outspoken difference between these groups was an almost twofold higher VWF activation factor (3.2 vs. 1.4; p<0.01) at enrollment in the children who died. No significant differences in these parameters were found between those with and those without clinical bleeding (data not shown). Infusion of blood products had only a minor influence on these median laboratory values because only one of the six children who died received FFP and platelets at the day of enrollment.

Correlations of VWF:Ag and VWF-related parameters with clinical and laboratory markers are shown in table 2. There was no significant correlation between VWF:Ag and markers of dengue severity (platelets and SGOT levels) and plasma leakage (albumin levels and PEI). In contrast, VWF propeptide levels had a much stronger correlation with these parameters; VWF propeptide levels were positively associated with severity of plasma leakage (negative correlation with plasma albumin level and positive correlation with PEI) and with liver enzyme disturbances and negatively associated with platelet count.

**Table 2:** Spearman correlation (r) of VWF-related variables with laboratory and clinical parameters of dengue severity.

	VWF:Ag		VWF propeptide		VWF activation factor		ADAMTS-13	
	r	*P*	r	*P*	r	*P*	r	*P*
VWF;Ag			0.28	0.15	**0.26**	**0.03**	−0.03	0.83
VWF propeptide	0.28	0.15			0.22	0.06	−0.22	0.06
VWF activation	**0.26**	**0.03**	0.22	0.06			−0.13	0.29
ADAMTS-13	−0.03	0.83	−0.22	0.06	−0.13	0.29		
Platelets	−0.06	0.61	**−0.41**	**<0.01**	−0.20	0.09	0.21	0.08
Albumin	−0.05	0.71	**−0.40**	**<0.01**	−0.20	0.10	**0.44**	**<0.01**
PEI	−0.25	0.06	**0.38**	**<0.01**	−0.15	0.25	−0.20	0.13
SGOT	0.01	0.94	**0.36**	**<0.01**	0.04	0.71	−0.23	0.06

Significant (*P*<0.05) correlations are shown in bold. VWF:Ag, von Willebrand factor antigen; PEI, pleural effusion index; SGOT, serum glutamic oxaloacetic transaminase.

## Discussion

Our study shows that DHF/DSS is associated with acute endothelial cell activation with exocytosis of WPBs and release of VWF:Ag, VWF propeptide and OPG in the circulation, combined with a decrease in ADAMTS-13 activity. The circulating VWF had a higher activation factor, indicating that an increased amount of VWF was in an elongated, ‘active’ conformation enabling spontaneous platelet-VWF binding. The patients who died had a significantly higher amount of VWF in its most active conformation and in some of the patients with low ADAMTS-13 activity at enrollment, qualitative defects in VWF with a pronounced loss of larger VWF multimers was seen in the discharge blood samples.

There was a clear difference in the kinetics of VWF:Ag and the other WPB constituents VWF propeptide and OPG: VWF:Ag levels were relatively low at enrollment and increased towards discharge, while VWF propeptide and OPG levels were very high at baseline and decreased upon clinical recovery. In contrast to VWF propeptide levels, VWF:Ag levels did not correlate well with parameters for disease severity. Hence, VWF propeptide levels seem to better reflect endothelial cell activation status and disease severity in DHF/DSS than VWF:Ag levels. We hypothesize that increased VWF consumption due to agglutinating platelets underlies this phenomenon. It is unlikely that VWF would simply leak out of the circulation during plasma leakage, just like albumin, because of the very large size of VWF multimers (>10.000 kDA) and because plasma levels of VWF propeptide would be expected to leak out even more as it is a smaller molecule. The four- to fivefold shorter half-life of VWF propeptide compared to mature VWF could explain why VWF:Ag levels were still elevated in the discharge samples, while VWF propeptide levels had returned to normal [2]. The VWF activation factor remained elevated across the study period. VWF activation is a measure of the relative amount of VWF that circulates in a platelet binding conformation. This parameter does not take into account the circulating VWF concentration, but total active VWF levels can be approximated by multiplying the VWF activation factor by the VWF:Ag levels. This approximation is hampered in this study by the consumption of VWF. Nonetheless, the high VWF propeptide levels in the early phase after enrollment and the fact that VWF:Ag and VWF propeptide are released in equimolar amounts from the endothelium suggest that total active VWF levels were higher early after enrollment and decreased towards discharge.

Our study is the first to report OPG data in dengue. The vascular effects of OPG have received increased attention in recent years. High OPG levels have been related to atherosclerosis and cardiovascular disease in epidemiological studies [22], [23] and OPG was shown to have specific effects on endothelial cells in vitro, such as prevention of apoptosis and up-regulation of adhesion molecules [8]–[10]. OPG is also closely linked to VWF: both proteins are cohabitants of WPBs and remain associated after release in the circulation. The notion that OPG binds selectively to the VWF A1 domain suggests that it may interfere with the binding of platelets to activated VWF, thereby preventing excessive platelet adhesion and aggregation [11], [24]. This process may especially be relevant in inflammatory conditions, which are usually accompanied by endothelial cell activation and release of VWF, since inflammatory cytokines up-regulate the synthesis and release of OPG [7]. OPG may also influence dengue pathogenesis in other ways. OPG is a decoy receptor that competes with both tumor necrosis factor-related apoptosis-inducing ligand (TRAIL) and receptor activator of nuclear factor kappa-B (RANK) ligand (RANKL). Patients with an acute dengue infection have elevated TRAIL serum levels [25]. This may be important in host defense against dengue, because recent work by Warke et al. suggested that TRAIL has dengue antiviral properties and suppresses the production of pro-inflammatory mediators by dengue-virus infected dendritic cells [26]. Moreover, while the RANK/RANKL system is predominantly known for its role in bone remodeling, there is increasing evidence that RANKL, which is among others expressed by activated T-cells, is also involved in regulation of immunity [27]. Hence, the high OPG levels may not only interfere with VWF-platelet interaction, but also with the antiviral and immune effects of TRAIL and the RANK/RANKL system.

While OPG levels increased during the acute phase of DHF/DSS, a reduction in ADAMTS-13 activity was common. ADAMTS13 regulates the multimeric size and function of VWF by cleaving VWF within the A2 domain. To our knowledge, no previous study has so far reported data on both OPG and ADAMTS13 in clinical samples. An acute increase in circulating VWF levels in volunteers through administration of desmopressin or endotoxin is followed by a decrease in ADAMTS-13 levels [28], [29] and lower ADAMTS-13 levels have been observed in several physiologic and pathologic conditions with high VWF levels, e.g. pregnancy and acute inflammatory states [30]. Hence, in our opinion, endothelial cell activation with release of VWF and secondary ADAMTS-13 consumption is the most likely explanation for the low ADAMTS-13 activity levels in these children. Other factors may also be involved: in vitro studies have shown that pro-inflammatory cytokines can reduce ADAMTS-13 synthesis, while thrombin and plasmin can inactivate ADAMTS-13 [31], [32]. Finally, a case of dengue-associated microangiopathic thrombocytopenia due to an inhibitor of ADAMTS-13 was recently reported [33]. The rapid normalization of ADAMTS-13 levels in the children in our study, however, argues against a role for ADAMTS-13 antibodies.

A remarkable observation in our study was the absence of large and intermediate VWF multimers in the discharge blood sample of some of the children. Loss of larger multimers is a prominent feature of acquired type 2A von Willebrand disease (VWD). This condition is associated with a number of different disease states, including hematoproliferative and auto-immune diseases and cardiac abnormalities such as aortic stenosis [34]. One case report described a transient acquired VWD in a child recovering from an acute EBV infection [35]. Different pathophysiologic mechanisms may underly the loss of larger multimers [34]. In essential and reactive thrombocytosis, the increased number of circulating platelets is associated with a reduction of large VWF multimers [36]. In dengue, platelet numbers start to rise abruptly in the convalescent phase and we hypothesize that the combination of rapidly rising platelet numbers, the restoration of ADAMTS-13 activity and ‘exhaustion’ of endothelial cells is responsible for the transient disappearance of large and intermediate VWF multimers. Loss of larger VWF multimers may result in an increased bleeding tendency and, although clinical bleeding in DHF/DSS is usually restricted to the critical phase around defervescence, qualitative abnormalities of VWF should be considered in patients with a persistent bleeding tendency.

Is the observed imbalance in the VWF-ADAMTS-13 system clinically relevant? High circulating levels of VWF in an elongated, active conformation together with reduced VWF proteolysis by ADAMTS-13 may lead to increased platelet adhesion and formation of platelet-rich thrombi. In patients with severe sepsis and severe malaria, a similar imbalance in VWF and ADAMTS-13 was found and this was considered to be related to thrombocytopenia and organ dysfunction [17], [37]–[40]. What DHF/DSS distinguishes from these other severe infectious diseases is that consumption of VWF;Ag and loss of larger VWF multimers have not been observed in these other diseases [17], [41], [42]. The notion that the AB blood group is associated with a higher risk for severe dengue supports a role for VWF in dengue pathogenesis, since blood group AB is associated with higher VWF levels [43]. Hence, even though typical features of thrombotic microangiopathy (e.g. schizocytes) are usually not found in dengue and the etiology of dengue-associated thrombocytopenia is multifactorial, we suggest that the observed changes in VWF-ADAMTS-13 play a role in the pathogenesis of DHF/DSS and in the etiology of thrombocytopenia in special. Disturbances in normal platelet-endothelium interaction may especially be relevant for DHF/DSS, since increasing evidence has shown platelet-endothelium interaction to be important for vessel wall stability during inflammation [44]. There is currently no specific treatment for DHF/DSS except careful fluid therapy. Although there is little evidence to support the practice of transfusing fresh-frozen plasma (FFP), this is sometimes done in practice for severe bleeding or for correction of prolonged coagulation tests. An unintended advantage of FFP infusion might be the replenishment of ADAMTS-13. One small trial in patients with acute dengue from Sri Lanka indeed found a small increase in platelet count in patients treated with 600 ml of FFP compared with isotonic saline [45].

Several limitations to our study should be considered. First, enrollment to our study was restricted to children with suspected DHF/DSS and samples from children with a mild dengue infection and uncomplicated dengue fever were not available for analysis. Whether the reported abnormalities in the VWF-ADAMTS13 system are specific for DHF/DSS or can also be found in less severe dengue infections therefore remains unknown. Second, platelet counts at discharge and in the control group were unavailable.

In conclusion, our data show that WPB exocytosis of VWF in its active conformation and consumption of VWF and ADAMTS-13 are prominent phenomena in severe dengue, which may contribute to thrombocytopenia and organ dysfunction. Severe dengue is also associated with very high plasma OPG levels, of which the functional consequences need further study. Finally, a transient reduction in larger VWF multimers may develop during convalescence.

## Supporting Information

Checklist S1 STROBE Checklist.
(DOCX)
